# Combinatorial Antitumor Effect of Rapamycin and *β*-Elemene in Follicular Thyroid Cancer Cells

**DOI:** 10.1155/2016/6723807

**Published:** 2016-05-04

**Authors:** Jun Zhou, Li-Li He, Xiao-Fei Ding, Qiu-Qi Yuan, Jian-Xin Zhang, Shuang-Chun Liu, Guang Chen

**Affiliations:** ^1^School of Medicine, Taizhou University, Taizhou, Zhejiang 318000, China; ^2^Taizhou Enze Hospital, Taizhou, Zhejiang 318000, China; ^3^Taizhou Municipal Hospital, Taizhou, Zhejiang 318000, China; ^4^School of Pharmaceutical and Chemical Engineering, Taizhou University, Taizhou, Zhejiang 318000, China; ^5^Institute of Tumor, Taizhou University, Taizhou, Zhejiang 318000, China

## Abstract

*Background.* mTOR signaling would be a promising target for thyroid cancer therapy. However, in clinical trials, objective response rate with mTOR inhibitor monotherapy in most cancer types was modest. A new focus on development of combinatorial strategies with rapalogs is increasing.* Objective.* Investigating the combinatorial antitumor effect of rapamycin and *β*-elemene in follicular thyroid cancer cells.* Methods.* MTT assay was used to determine the FTC-133 cell proliferation after culturing with rapamycin and/or *β*-elemene. To analyze their combinatorial effect, immunoblotting was performed to analyze the activation status of AKT. Moreover, *β*-elemene attenuated rapamycin-induced immunosuppression was tested in mice.* Results.* Combination of rapamycin and *β*-elemene exerted significant synergistic antiproliferative effects in FTC-133 cell lines* in vitro*, based on inhibiting the AKT feedback activation induced by rapamycin.* In vivo*, the *β*-elemene could attenuate rapamycin-induced immunosuppression via reversing imbalance of Treg/Th17, with the underlying mechanism needed to be declared.* Conclusions*. We demonstrate that the novel combination of mTOR inhibitor with *β*-elemene synergistically attenuates tumor cell growth in follicular thyroid cancer, which requires additional preclinical validation.

## 1. Introduction

Follicular thyroid cancer is the second most common cancer of the thyroid after papillary carcinoma. It accounts for 15% of thyroid cancer and occurs more commonly in women over 50 years of age. Despite its well-differentiated characteristics, follicular carcinoma may be overtly or minimally invasive. In fact, FTC tumors may spread easily to other organs [1]. For the advanced follicular thyroid carcinoma, the targeted drug therapy is important. It has been reported that mTOR signaling would be a promising target for thyroid cancer therapy [2–5].

The mTOR is a serine/threonine kinase and downstream member of the phosphatidylinositol-3-kinase (PI3K) pathway and plays an important role in regulating many physiological and pathological conditions, such as cell proliferation, angiogenesis, metabolism, differentiation, and survival [6, 7]. The mTOR-mediated pathway is widely implicated in cancer and has been validated as an oncotarget. Rapalogs are recognized as the first generation of mTOR inhibitors, which inhibit the activity of mTORC1 via binding to FKBP-12 and forming a ternary complex with mTOR. Temsirolimus and everolimus were approved by FDA for the treatment of patients with advanced renal cell carcinoma. However in a phase II trial using a traditional approach in breast cancer, objective response rate with temsirolimus monotherapy was only 9.2%. That is consistent with results that objective response rates with rapalogs were modest in most tumor types and highly variable. One important strategy to improve the efficacy of rapalogs is development of combination regimens [8].

The *β*-elemene is the essential oil extracted from* Curcuma wenyujin*, Y. H. Chen et C. Ling, a traditional Chinese herb medicine. The *β*-elemene has been shown to inhibit tumor cell growth and elemene emulsion has been approved for cancer treatment [9–13]. However, objective response rate with *β*-elemene was also modest in most tumor types. Combining therapy with mTOR inhibitor and *β*-elemene may overcome the resistance of some tumors to mTOR inhibitor or *β*-elemene. Furthermore, the ability to modulate the immune system of *β*-elemene may overcome the immunosuppression induced by mTOR inhibitor as an immunosuppressant drug.

Here, we showed that the cotreatment with rapamycin and *β*-elemene is effective against follicular thyroid carcinoma.

## 2. Materials and Methods

### 2.1. Cell Culture and Reagents

FTC-133 cells were obtained from CULTURE CELLECTIONS (Public Health England, Porton Down, UK) and maintained in appropriate medium as suggested by CULTURE CELLECTIONS (DMEM : Ham's F12 (1 : 1) + 2 mM Glutamine + 10% Foetal Bovine Serum). Cells were incubated in a humidified atmosphere of 95% air plus 5% CO_2_ at 37°C. The *β*-elemene was obtained from Dalian Holey Kingkong Pharmaceutical Co., Ltd. (Dalian, China). Rapamycin was obtained from LC Laboratories (Woburn, MA, USA). DMSO is the solvent used for making rapamycin and beta-elemene* in vitro*.

### 2.2. MTT Assay

Cell proliferation was evaluated by 3-(4,5-dimethylthiazol-2yl)-2,5-diphenyltetrazolium bromide (MTT) assay. Briefly, 5000 cells were seeded into 96-well plates and cultured overnight and treated with rapamycin and/or *β*-elemene for another 72 h. At the end of the 72 h incubation period, the reaction was terminated by adding MTT to each well with final concentration of 0.5 mg/mL. The reaction was allowed to proceed for 3~4 hours at 37°C. The formazan crystals were then dissolved by adding 0.1 mL of dimethyl sulfoxide (DMSO). The intensity of the color developed, which is the reflection of number of live cells, was measured at 490 nm using a multiwell spectrophotometer. All values were compared to the corresponding controls. All assays were performed with 3 replicates. The inhibition rate on cell proliferation was calculated for each well as (*A*_490,control  cells_ − *A*_490,treated  cells_)/*A*_490,control  cells_ × 100% (*A*_490_: OD value at 490 nm).

### 2.3. Transwell Assay

Cell migration was evaluated using an 8 mm pore size Transwell system (Costar, Cambridge, MA, USA). Briefly, cells were resuspended in serum-free RPIM-1640 at a density of 2 × 10^5^ cells/mL. The top chamber of Transwell was loaded with 100 *μ*L of cell suspension and candidate compound, while the bottom chamber was loaded with 0.6 mL of RPIM-1640 containing 10% FBS and candidate compound. The total migrated cells to the lower chamber were fixed, stained with 0.1% crystal violet, and photographed after treatment. Crystal violet stained cells were dissolved with 10% acetic acid and OD value was measured at 595 nm.

### 2.4. Immunoblotting

Immunoblotting was conducted with standard procedures, using antibodies against AKT and phosphorylated AKT (Cell Signaling Technology, Beverly, MA).

### 2.5. Animals

Seven-week-old specific pathogen-free male Kunming mice (weight, 18–22 g) were supplied by the Laboratory Animal Center of Zhejiang Academy of Medical Sciences. Solvent control (p.o.), rapamycin (p.o.), and *β*-elemene (i.p.) were administered for 7 days. After treatment, animals were killed by cervical dissociation. The thymus and spleen were removed and weighed.

All animals used in the study were housed and cared for in accordance with the Chinese Pharmacological Society Guidelines for Animal Use. The work was approved by the Committee on the Ethics of Animal Experiments of the Taizhou University (Permit number: 2014-019). All surgery was performed under sodium pentobarbital anesthesia, and all efforts were made to minimize suffering.

### 2.6. Isolation of Spleen Lymphocytes

The spleen was surgically removed and prepared by gently pressing the organs through a 74 *μ*m filter and the cells were then washed and the red cells were lysed. CD4^+^ CD25^+^ Foxp3^+^ Treg, CD4^+^ IL-17^+^ Th17, and CD4^+^ cells from splenocytes were counted by using FACS (Attune, Life Technologies).

## 3. Result

### 3.1. Combinatorial Antitumor Effect of Rapamycin and *β*-Elemene on FTC-133 Cells Proliferation and Migration

**Figure 1:**
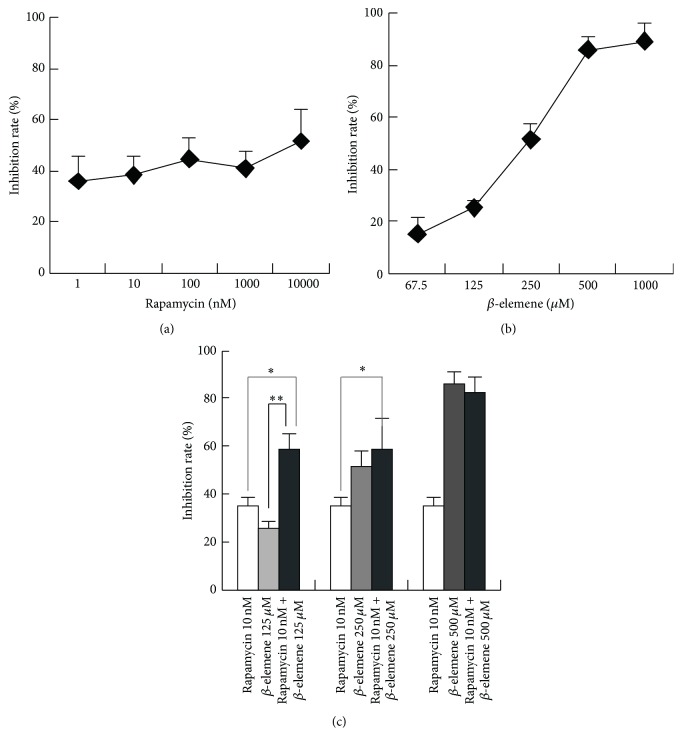
Antitumor activity of rapamycin or/and *β*-elemene* in vitro*. (a) Antiproliferation activity of rapamycin in FTC-133 cells. (b) Antiproliferation activity of rapamycin in FTC-133 cells. (c) Combinatorial antitumor effect of rapamycin and *β*-elemene on FTC-133 cells proliferation. Cells were treated with various concentrations of rapamycin or/and *β*-elemene for 72 h. Cell viability was determined by MTT assay. Columns, mean inhibition rate (%) of three independent experiments; bars, SD. ^*∗*^ *p* < 0.05 versus rapamycin group and ^*∗∗*^ *p* < 0.01 versus *β*-elemene group.

MTT assay was used to determine the FTC-133 cell growth after culturing with rapamycin and/or *β*-elemene for 3 days. Data in Figure 1 shows that treatment of FTC-133 cells with rapamycin alone (1 nM–10 *μ*M) resulted in an inhibition in cell proliferation, with inhibition rates ~40% that followed a dose-independent manner. *β*-elemene displayed potent proliferation inhibition activity in a dose-dependent manner in FTC-133 cell, with IC50 values of 266.1 ± 9.5 *μ*M. On the other hand, the combination of rapamycin plus *β*-elemene caused a significant inhibition in cell proliferation that followed a synergistic action in a concentration of 10 nM rapamycin and 125 *μ*M *β*-elemene, with the coefficient of drug interaction (CDI) being 0.86.

**Figure 2:**
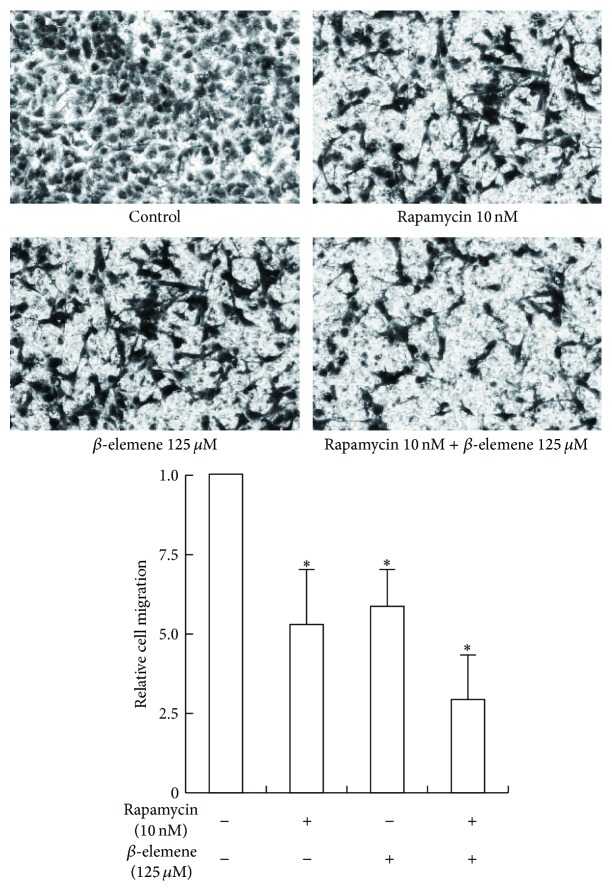
Combinatorial antitumor effect of rapamycin and *β*-elemene on FTC-133 cells migration. The experiments above were conducted thrice. Columns indicate the mean of three experiments; bars, SD. ^*∗*^ *p* < 0.01 versus control group.

To validate the combinatorial antitumor effect of rapamycin and *β*-elemene, we detected the migration of rapamycin and/or *β*-elemene treatment FTC-133 cells. Rapamycin combined with *β*-elemene caused an obvious inhibition in cell migration, with more effective activities than each single drug treatment (Figure 2).

### 3.2. *β*-Elemene Attenuated Rapamycin-Induced AKT Activation

It is well known that rapamycin mediates AKT activation through an IGF-1R-dependent mechanism [14–16], which is related to rapamycin resistance in cancer [17, 18]. However the antitumor effect of *β*-elemene is associated with the inhibition of MAPK/ERK and PI3K/AKT/mTOR signaling pathway [19]. To analyze the combinatorial effect of rapamycin and *β*-elemene, western blotting was performed to analyze the activation status of AKT.

**Figure 3:**
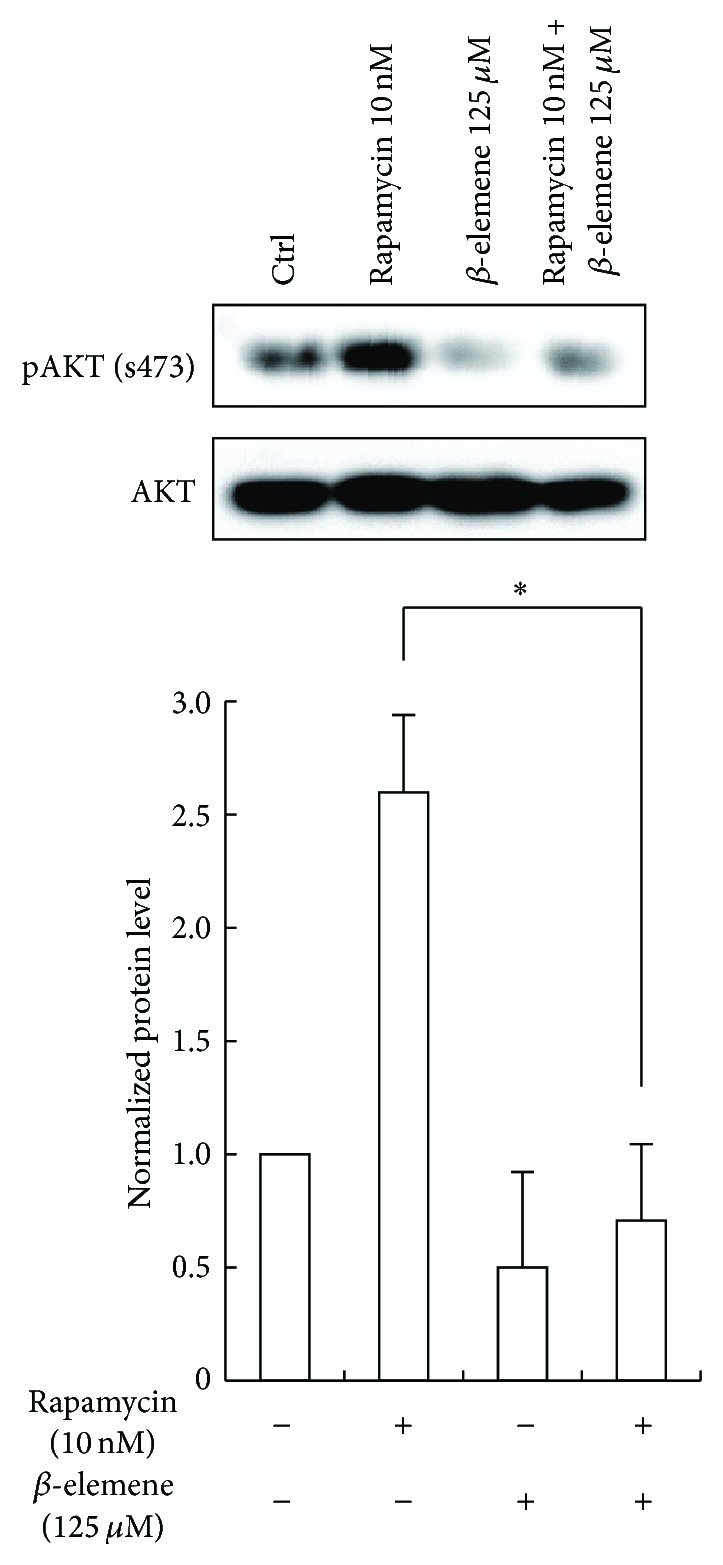
*β*-elemene attenuated rapamycin-induced AKT activation. Phosphorylated AKT was analyzed by immunoblotting. Total AKT was employed as a control. The experiment was repeated at least thrice. ^*∗*^ *p* < 0.01 versus rapamycin group.

As shown in Figure 3, the level of phosphorylated AKT at S473 was upregulated obviously in the FTC-133 cell treatment with rapamycin 10 nM for 24 hours. On the other hand, the *β*-elemene 125 *μ*M treatment for 24 hours could significantly decrease the level of phosphorylated AKT at S473. Moreover, *β*-elemene could also attenuate rapamycin-induced AKT activation. It seemed that *β*-elemene sensitizes FTC-133 cell to rapamycin via inhibition of the AKT feedback activation.

### 3.3. *β*-Elemene Attenuated Rapamycin-Induced Immunosuppression

As an immunosuppressive drug, rapamycin has been used clinically to prevent graft rejection for sixteen years because of its suppressive effects on T cell activation and proliferation. But, as antitumor drugs, rapalogs (temsirolimus and everolimus) were approved by FDA only for six years. So rapalogs would cause tumor patient immunosuppression while the *β*-elemene is a natural antitumor plant drug, which is strongly implicated as an immune modulatory agent. Combining rapamycin with *β*-elemene would attenuate rapamycin-induced immunosuppression.

**Figure 4:**
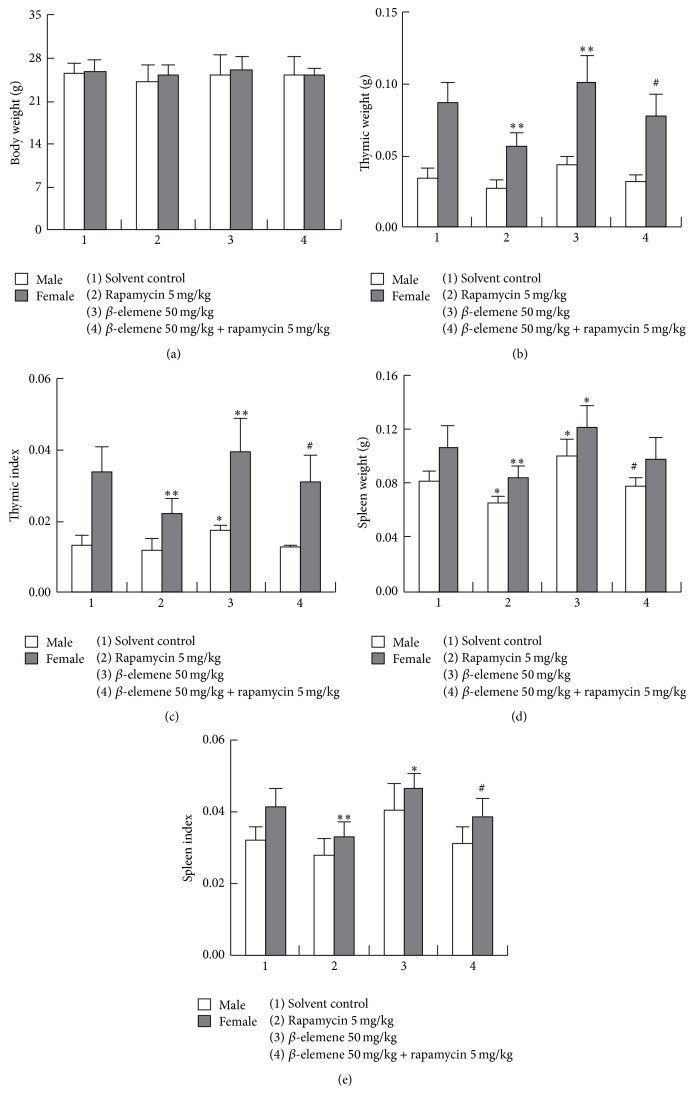
*β*-elemene attenuated rapamycin-induced immunosuppression. Data shown are mean ± SD, *n* = 10. ^*∗*^ *p* < 0.05 and ^*∗∗*^ *p* < 0.01 versus control group and ^#^ *p* < 0.05 versus rapamycin group.

As shown in Figures 4(b), 4(c), and 4(d), rapamycin 5 mg/kg p.o. for 7 days, the thymus weight, thymus index, spleen, and spleen index of female mice were decreased significantly. However, in the male mice, just the spleen weight decreased obviously. It seemed that rapamycin showed more potential immunosuppression in female mice than male mice; the underlying mechanism is still to be spelled out. However, *β*-elemene could attenuate rapamycin-induced immunosuppression.

### 3.4. *β*-Elemene Reserved Rapamycin-Induced Imbalance of Treg/Th17 in Mice

Cancer patients usually exhibit Th17/Treg balance disorders with higher Treg and lower Th17 cells. It indicates that Th17/Treg balance disorder plays an important role in the discovery and development of cancer [20–23]. Based on the FCM analysis (Table 1), we found that the ratio of Treg/Th17 was increased significantly after rapamycin treatment, which is consistent with the previous studies [24]. However, in *β*-elemene treatment group the ratio was decreased obviously compared with control group, which suggested that the Treg/Th17 balance tilted to Th17. Meanwhile, *β*-elemene could significantly attenuate rapamycin-induced increase of Treg/Th17 ratio.

## 4. Discussion

In the present study, we investigated the effect of a combination of mTOR inhibitor (rapamycin) and a natural antitumor plant drug (*β*-elemene) on follicular thyroid cancer. We found that they exerted significant synergistic antiproliferative effects in FTC-133 cell lines* in vitro*. This combination worked via inhibiting the AKT feedback activation induced by rapamycin. We also demonstrated that the *β*-elemene could attenuate rapamycin-induced immunosuppression.

mTOR inhibition as an anticancer strategy has been previously tested, and rapalogs (temsirolimus and everolimus) were approved by FDA for the treatment of patients with advanced renal cell carcinoma, following the positive results of phase III trials. Although rapalogs have shown preclinical promise, their roles as single agents for other cancer types in phase 2 studies have resulted in only modest responses. It suggested that mTOR inhibitors will have maximum benefit when rationally combined with other agents, based on inhibiting multiple signaling cascades, such as IGFR inhibitor figitumumab [25], EGFR inhibitors gefitinib [26], and ER inhibitors [27]. Our data support the notion that combination therapy that ablates mTOR function and prevents AKT activation may have improved antitumor activity. We show that the combination of *β*-elemene and rapamycin overcomes the feedback activation of AKT. 10 nM rapamycin plus 125 *μ*M *β*-elemene resulted in synergistic inhibition of proliferation in FTC-133 cells.

Although rapamycin dose for tumor treatment is a little lower than that used for immunosuppression in transplantation, mTOR inhibitors still have the potential in immunosuppression. Here, we showed that the immune modulatory agent *β*-elemene attenuated rapamycin-induced immunosuppression, with the underlying mechanism of restoring the Th17/Treg balance disorder induced by rapamycin treatment.

In conclusion, we found that the combination of *β*-elemene and rapamycin exerted significant synergistic antiproliferative effects in FTC-133 cell lines* in vitro*, based on preventing AKT feedback activation. We also show that they could overcome rapamycin-induced immunosuppression; the underlying mechanism needs to be further declared. Our primary findings provide a rationale to conduct preclinical or clinical trials with mTOR inhibitor and *β*-elemene in follicular thyroid cancer patients.

## Figures and Tables

**Table 1:** Treatment of *β*-elemene could reverse the imbalance of Treg/Th17 in the mouse spleen induced by rapamycin (mean ± SD, *n* = 6).

	Group			
	Control	Rapamycin 5 mg/kg	*β*-elemene 50 mg/kg	Rapamycin 5 mg/kg + *β*-elemene 50 mg/kg
Treg/Th17	2.2 ± 0.2	5.8 ± 0.6^#^	1.1 ± 0.3^#^	3.4 ± 1.1^*∗*^
Treg/CD4^+^ (%)	7.6 ± 0.8	12.2 ± 1.3^#^	4.7 ± 0.7^#^	10.1 ± 2.8^*∗*^
Th17/CD4^+^ (%)	3.6 ± 0.4	2.1 ± 0.2^#^	4.4 ± 1.3	2.8 ± 0.9
CD4^+^ T (%)	25.4 ± 3.1	22.9 ± 4.3	27.7 ± 4.5	26.6 ± 5.3

^#^ *p* < 0.05  versus control group; ^*∗*^ *p* < 0.05  versus rapamycin group.
