# Correction to “Combinatorial Antitumor Effect of Rapamycin and *β*‐Elemene in Follicular Thyroid Cancer Cells”

**DOI:** 10.1155/bmri/9791483

**Published:** 2026-09-10

J. Zhou, L.‐L. He, X.‐F. Ding, Q.‐Q. Yuan, J.‐X. Zhang, S.‐C. Liu, and G. Chen, "Combinatorial Antitumor Effect of Rapamycin and *β*‐Elemene in Follicular Thyroid Cancer Cells," BioMed Research International 2016, no. 1 (2016): 1–8, https://doi.org/10.1155/2016/6723807.

In the article titled “Combinatorial Antitumor Effect of Rapamycin and *β*‐Elemene in Follicular Thyroid Cancer Cells” there are errors in Figure 2. The images presented were incorrectly taken from another study conducted in parallel by the authors [1]. The overlap occurred as both projects focused on the same mTOR (mammalian target of rapamycin) pathway and utilized similar experimental protocols. The errors occurred during image assembly.

Additionally, the authors would like to correct the associated statistics analysis by further differentiating the significance levels presented, using ‘ ^∗∗^p <0.01’, and introducing ‘ ^∗∗∗^p <0.001’. This provides a more accurate representation of the statistical strength based on the data and supports the original conclusions.

Figure 2 should be corrected as follows:

**Figure 2:**
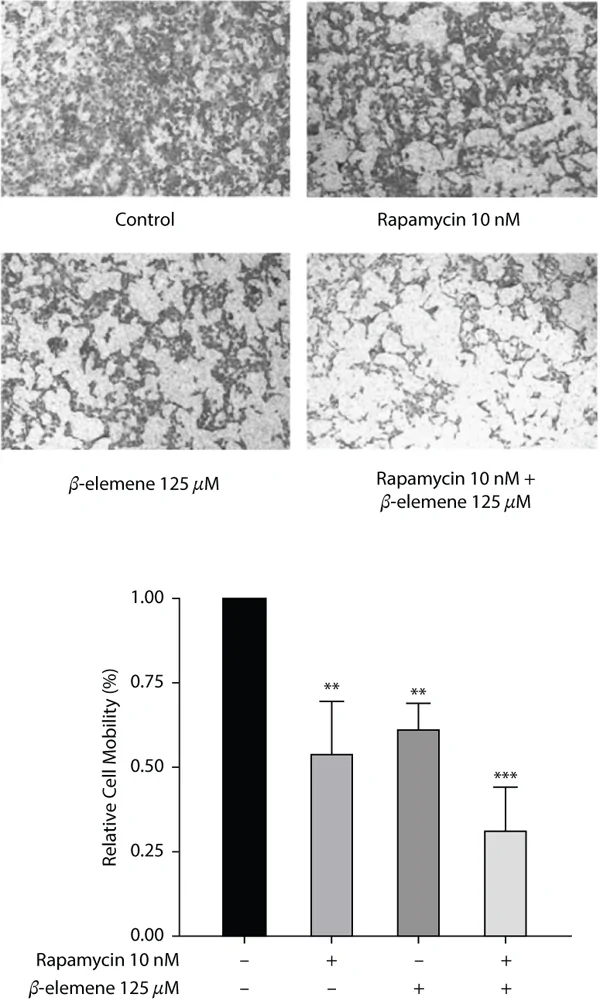
Combinatorial antitumor effect of rapamycin and *β*‐elemene on FTC‐133 cells migration. The experiments above were conducted thrice. Columns indicate the mean of three experiments; bars, SD.  ^∗∗^ *p* < 0.01,  ^∗∗∗^ *p* < 0.001 versus control group.

We apologize for these errors.
